# A Mobile Social Network–Based Smoking Cessation Intervention for Chinese Male Smokers: Protocol for a Pilot Randomized Controlled Trial

**DOI:** 10.2196/18071

**Published:** 2020-09-18

**Authors:** Jinsong Chen, Elsie Ho, Yannan Jiang, Robyn Whittaker, Tingzhong Yang, Christopher Bullen

**Affiliations:** 1 The National Institute for Health Innovation The University of Auckland Auckland New Zealand; 2 School of Population Health The University of Auckland Auckland New Zealand; 3 Centre for Tobacco Control Research School of Medicine The Zhejiang University Hangzhou China

**Keywords:** mHealth, mobile phone, smoking cessation, public health

## Abstract

**Background:**

Approximately 2 million Chinese people die annually from tobacco-related diseases, mostly men; yet, fewer than 8% of Chinese smokers ever receive any smoking cessation advice or support. A social network–based gamified smoking cessation intervention (*SCAMPI: Smoking Cessation App for Chinese Male: Pilot Intervention*) is designed to help Chinese male smokers to quit smoking.

**Objective:**

This paper aims to present the protocol of a study examining the preliminary effectiveness of SCAMPI by comparing the prolonged abstinence rate of a group of users with a comparator group during a 6-week follow-up period.

**Methods:**

A two-arm pilot randomized controlled trial was conducted to assess the preliminary effectiveness and acceptability of the SCAMPI program as a smoking cessation intervention. After initial web-based screening, the first 80 eligible individuals who had gone through the required registration process were registered as participants of the trial. Participants were randomly allocated to the intervention group (n=40) and the control group (n=40). Participants in the intervention group used the full version of the SCAMPI program, which is a Chinese smoking cessation program developed based on the Behavior Change Wheel framework and relevant smoking cessation and design guidelines with involvement of target users. The program delivers a range of smoking cessation approaches, including helping users to make quitting plans, calculator to record quitting benefits, calendar to record progress, gamification to facilitate quitting, providing information about smoking harms, motivational messages to help users overcome urges, providing standardized tests to users for assessing their levels of nicotine dependence and lung health, and providing a platform to encourage social support between users. Participants in the control group used the restricted version of the SCAMPI program (*placebo app*).

**Results:**

Recruitment for this project commenced in January 2019 and proceeded until March 2019. Follow-up data collection was commenced and completed by June 2019. The primary outcome measure of the study was the 30-day bio-verified smoking abstinence at the 6-week follow-up (self-reported data verified by the Nicotine Cotinine Saliva Test). The secondary outcome measures of the study included participants’ cigarette consumption reduction (compared baseline daily cigarette consumption with end-of-trial daily cigarette consumption), participants’ 7-day smoking abstinence at 4-week and 6-week follow-up (self-reported), participants’ 30-day smoking abstinence at 6-week follow-up (self-reported data only), and participants’ acceptability and satisfaction levels of using the SCAMPI program (measured by the Mobile App Rating Scale questionnaire).

**Conclusions:**

If the SCAMPI program is shown to be preliminary effective, the study will be rolled out to be a future trial with a larger sample size and longer follow-up (6 months) to identify if it is an effective social network–based tool to support Chinese male smokers to quit smoking.

**Trial Registration:**

Australian New Zealand Clinical Trials Registry ACTRN12618001089224; https://www.anzctr.org.au/Trial/Registration/TrialReview.aspx?id=375381

**International Registered Report Identifier (IRRID):**

RR1-10.2196/18071

## Introduction

### Burden of Tobacco Smoke

Every year, approximately 2 million Chinese people are killed by tobacco-caused diseases [1]. Still, nearly 300 million Chinese people continue to use tobacco each day [1]. Among these smokers, 95% are male [2]. Tobacco use caused 25% of Chinese male deaths [1]. In addition to health harm, tobacco use in China also leads to societal harm [1]. The economic cost of smoking in China amounts to ¥393 billion yuan (US $57.4 billion dollars) per year [1]. This includes direct costs related to health care expenditures and indirect costs related to lost productivity due to early mortality and morbidity [1].

According to the 2010 Global Adult Tobacco Survey in China, 36.4% of Chinese smokers had tried to quit in the past 12 months, whereas 91.8% of them had never received any smoking cessation services [3]. A survey in 2014 found that over half of Chinese smokers never received any quit smoking advice from their health professionals [4]. Since 2006, although some smoking cessation clinics had been set up and the National Quitline had launched, due to limited exposure and accessibility, they have rarely been used by Chinese smokers [5].

### Mobile Smoking Cessation

A 2019 Cochrane review of mobile phone–based interventions for smoking cessation demonstrated the effectiveness of mobile technology in supporting smoking cessation [6]. The most recent World Health Organization report on the global tobacco epidemic indicated that personalized smoking cessation advice and support from mobile phone messages can be a cost-effective tool contributing to address the public health problem of tobacco use [7]. Mobile phone–based smoking cessation interventions have proven to be a cost-effective tool for supporting smokers to quit smoking. Some studies also showed that mobile smoking cessation apps can create short-term impacts on helping smokers to quit smoking [8-13]. A recent study has shown that daily smokers using a behavioral, decision-aid smartphone app achieved 23.8% self-reported 3-month continuous abstinence [14].

The 2017 Connected Consumer Survey conducted by Google Inc noted that 83% of Chinese people use smartphones [15]. China is regarded as the fastest growing smartphone market in the world, with a 10% increase in smartphone penetration rate in the past 5 years [16]. China has the largest smartphone user group (n>700 million people) in the world [17]. Among all the functions and apps of smartphones, WeChat is the most popular app (social network platform) in China. In 2020, 1.2 billion people were monthly active users of WeChat [18]. WeChat users are highly engaged with the app; nearly 80% of WeChat users use the app for >30 min daily [18]. WeChat has become a major tool in communication, entertainment, and payment for Chinese smartphone users.

### Aim of the Study

As previously mentioned, Chinese males are the largest smoking population group in the world. Most Chinese smokers have limited access to smoking cessation services. The lack of appropriate smoking cessation services and limited accessibility to current smoking cessation support lead to a low smoking cessation success rate [19]. The high smartphone penetration rate and usage rate in China provide a great opportunity for developing and implementing smartphone app–based smoking cessation interventions. The potential for WeChat-based smoking cessation interventions to impact and reach out to millions of Chinese smokers is possible like never before.

The prevalence of cigarette smoking in Chinese males is substantial, and mobile smoking cessation programs hold much promise to provide cost-effective support to help smokers to quit. To date, there have been no reported investigations on the efficacy of delivering a smartphone social network–based smoking cessation intervention for Chinese male smokers via a social network platform.

The aim of this study is to introduce the SCAMPI program, which was designed to support Chinese male smokers to quit smoking and present the protocol of a pilot randomized controlled trial (RCT) that assesses the preliminary effectiveness of the SCAMPI program. The study examines the quit rates of abstaining users of the full version SCAMPI program versus the quit rates of a comparator group for a 6-week period. The pilot trial’s main hypothesis is that users of the full version program will have higher biochemical verified 30-day smoking abstinence during the follow-up measures in comparison with the comparator group.

## Methods

### Study Design

This study will be a pilot, two-arm, parallel RCT. We will evaluate the preliminary effectiveness and acceptability of our SCAMPI program as well as recruitment and retention for estimating the sample size of a future definitive trial. All trial procedures will be conducted web-based via WeChat (Tencent). Ethical approval was granted by the University of Auckland Human Participants Ethics Committee (reference number: 021649) and the Zhejiang University School of Public Health Research Ethics Committee (reference number: ZGL201801-2).

### Study Setting

The study setting of this pilot trial will be completely online via the social network platform WeChat. The SCAMPI program will not need to be downloaded or installed on participants’ mobile phones, but they will be authorized to use the program via the WeChat platform.

### Participants

#### Eligibility Criteria

Chinese male smokers will be eligible for inclusion in the study if they indicate at screening that they are smokers (both daily smokers *smoking any types of tobacco products on a daily basis* or occasional smokers *smoking any types of tobacco products occasionally [at least once in a week]*) aged between 25 and 44 years [5], have access to a smartphone, have a WeChat account, have adequate knowledge of Chinese language, and are willing to participate in the study and provide follow-up information at scheduled points of the study. People will be excluded from the sample if they report in the screening test that they are involved in any other types of smoking cessation interventions.

#### Recruitment

Online advertisement of the pilot trial will be delivered through WeChat to different WeChat users. Potential participants who read the trial advertisement and were interested in participating could subscribe to the SCAMPI WeChat official account (OA). Once they subscribe to the account, they will receive an auto-replied trial instruction. People can tap the registration link on the instruction to register as a participant of the study.

In the registration system, potential participants will be screened to ascertain their eligibility for the study. Eligible participants will be provided with the participant information sheet and consent form for them to read. After they complete reading the documents, they will be asked to provide electronic consent (e-consent) by tapping the *agree* button.

Within the e-consent, participants will then be directed to complete the baseline assessment. A participant will receive his first study compensation once he completes the registration to strengthen his relationship with the study.

#### Randomization and Allocation

Participants that fulfilled eligibility criteria and completed baseline assessment will be randomized in a 1:1 ratio to either an intervention group or a control group. The randomization sequence was generated by the trial statistician (YJ) using block randomization with variable block sizes of 2 or 4. The final randomization list was concealed in the database until the point of randomization.

Randomization will be performed upon completion of the baseline assessment. Each participant will have a unique code based on the sequence of completing the registration (participant no. 1 to no. 80). Randomization will be implemented based on the participant’s code. Participants who are randomized into the intervention group will have access to the full version of the SCAMPI program. Participants who are randomized into the control group will have access to the restricted version of the SCAMPI program.

### Intervention

The SCAMPI program was designed using the behavior change wheel (BCW) framework, a theory- and evidence-based tool for designing interventions based on an analysis of the nature of the behavior, the mechanisms that need to be changed to bring about behavior change, and the interventions and policies required to change those mechanisms [20]. It is considered as one of the most inclusive behavior change framework that was developed from 19 frameworks of behavior change identified in a systematic literature review [20].

The BCW framework starts with a theoretical understanding of behavior by the *capability, opportunity, and motivation to behavior model* (COM-B model) to determine what needs to change for the behavioral target to be achieved and what intervention functions are likely to be effective to bring about that change [20]. After selecting the intervention functions most likely to be effective in changing the smoking behavior of the target population, these will then be linked to specific behavior change techniques (BCTs), which will be codable to a mobile social network–based smoking cessation program [21,22]. The content, user interface (UI), and user experience (UX) design of the SCAMPI program were developed based on the China Clinical Smoking Cessation Guideline (CCSCG) [23] and WeChat mini-program design guidelines [24].

The application of the BCW framework for developing the SCAMPI program was under a process called collaborative product development (CPD), which is based on the principles of user-centered design (UCD) developed by Preece et al in 2004 [25]. In this study, UCD occurred in 2 forms: (1) consulting users about their needs of the proposed mobile health app–based smoking cessation intervention and (2) involving users as partners with researchers throughout the design and development process [25].

In the CPD stage, 20 potential end users (Chinese male smokers aged 25 to 44 years) were recruited through WeChat. These 20 participants provided their ideas about the desired components of a smoking cessation program through completing a development questionnaire (Multimedia Appendix 1). In the 1-month period, app development progressed and relevant questions were posted on SCAMPI OA to identify participants’ preferences for the program’s UX and UI design. At the end of the CPD period, participants’ acceptability and satisfaction level toward the app were identified by completing an online questionnaire based on standard questions from the Mobile App Rating Scale (MARS) questionnaire (Multimedia Appendix 2) [26]. A total of 16 out of 20 participants completed the questionnaire, and the average rating of the SCAMPI program was 4.4 out of 5.0.

On the basis of the findings from participants’ responses to the development questionnaire and the behavior analysis of the target population’s smoking behavior [5,27], 11 key behavioral factors of the target population’s smoking behavior were summarized. These 11 factors reflect the 5 main components of the COM-B model. By applying the BCW to navigate the BCTs, 48 BCTs are relevant to these 5 reflected components of the COM-B model. Screening by the WeChat mini-program design guideline and the relevant context (eg, the program will be used by Chinese male smokers in China), 43 BCTs were considered as deliverable through the WeChat platform (Multimedia Appendix 3). Identified BCTs were integrated and coded as functions of the program. Table 1 shows the functions of the SCAMPI program and the corresponding BCTs that are aimed to be achieved by each function.

**Table 1:** Functions of the program and corresponding behavior change techniques codes.

Functions	Corresponding behavior change techniques
Design of the program	RD^a^1
Calculator, data capturing, and recoding	BS^b^6, BM^c^9, RC^d^8, RI^e^1, RI2, RI3, and RI4
Calendar	BM3
Content of the program	RC1, RC4, and RC7
Game	BM5, BS4, BS5, and BS6
Informational and practical tool	BM1, BM10, BS1, BS2, BS10, A^f^5, RC5, RC6, RC10, BM8, BS10, BS7, BS8, BS11, and RD2
Motivation	BM2
Other	Health testers, red packet (compensation)
Planning	BS3
Social support	A2

^a^RD: general aspects of the interaction (R) focusing on the delivery of the intervention (D).

^b^BS: specific focus on behavior (B) and maximizing self-regulatory capacity or skills (S).

^c^BM: specific focus on behavior (B) and addressing motivation (M).

^d^RC: general aspects of the interaction (R) focusing on general communication (C).

^e^RI: general aspects of the interaction (R) focusing on information gathering (I).

^f^A: promote adjuvant activities (A).

In summary, besides the design and content, the SCAMPI program has the following functions: (1) calculator to record quitting benefits (eg, money saved by not smoking), (2) calendar to record the progress of smoking cessation (eg, which date the users did not smoke), (3) gamification to facilitate quitting (eg, ranking board for showing users who make the longest continuous smoking abstinence), (4) providing information about smoking harms and health benefits from smoking cessation, (5) sending motivational messages (based on requests from users) to help users overcome urges, (6) providing standardized tests to users for assessing their levels of nicotine dependence and lung health, (7) helping users to make smoking cessation plans, and (8) providing a social support platform to users for delivering peer support between users. Screenshots and Quick Response (QR) codes of the SCAMPI program are provided in Multimedia Appendix 4.

As the target users of the program will be Chinese male smokers, all contents of the program were designed to be in Chinese. Table 2 presents examples of database content by themes (eg, smoking harms and quitting tips). The database content is based on the taxonomy of BCTs for smoking cessation via a text messaging intervention [22]. The program database includes a pool of 40 articles about smoking harms, all of which are coded to be sent to users on a daily basis through the SCAMPI OA. Each of these articles may take less than 3 min for participants to read. Articles are presented in the form of text and images. Participants will be prompted to read the articles, and they can decide when to read the articles and if they want to re-read the articles. All articles will be stored in SCAMPI OA throughout the trial and after the trial is completed. Participants in both groups and other subscribers to the SCAMPI OA will be able to have access to these articles freely after the completion of the trial. Information on the articles was referred to the CCSCG [23]. In addition, the program was designed according to the WeChat mini-program design guidelines [24]. The principles of friendliness and courtesy, clarity, convenience and elegance, and consistency have been used in the UX and UI design of the program.

**Table 2:** Examples of the program database content.

Themes	Examples of titles	Behavior change techniques
Smoking harms	“There are about 7,000 chemical components in tobacco, 69 of them are carcinogenic?!”	Provide information on consequences of behavior in general
Quitting tips	“When you feel urge to smoking, try deep breath and some physical exercise to distract your urge.”	Provide information on supporting behavior
Motivational messages	“Keep it up! You are XX days without smoking.”	Facilitate relapse prevention

Participants in the intervention group will have access to the full version of the SCAMPI program, and participants in the control group will have access to the restricted version of the SCAMPI program. Participants of the trial had free access to the SCAMPI program, both the full and the restricted versions. By the end of the trial, all WeChat users will have free access to the SCAMPI program. People can have access to the SCAMPI program by either search keywords “smoking or smoking cessation or SCAMPI” on the WeChat platform or scan the SCAMPI program QR codes.

The full version of the SCAMPI program has functions and content as described above. The restricted version of the program provides contact information of standard smoking cessation care (eg, Quitline in China and local smoking cessation clinics) to its users. Participants in both groups can use the corresponding versions of the SCAMPI program based on their willingness of when, where, and how long. The minimum frequency of using the program was once a week, to provide participants’ daily cigarette smoking status throughout the week.

One investigator (JC) will have a minimum level of involvement in the trial, only if any of the following situations happen: (1) participants develop serious mental health issues by quitting smoking, investigator will terminate the participation immediately and refer the participants to health care providers; (2) participants want to withdraw from the trial, investigator will kindly ask the reasons for withdrawal only if participants are willing to answer; and (3) participants do not provide their smoking status for a week, friendly reminders will be sent by the investigator to remind participants to provide the data.

Prompts and messages of the program will only be triggered by requests from users (eg, motivational messages to support users from smoking urges or read program posts about information on smoking harms). The only message users receive will be to remind them to provide their smoking status; it will be triggered in the 48 hours after the date that users are requested to provide their daily smoking status of the week. The reminding messages will be delivered at a frequency of 3 times a day (once every 8 hours) for 2 days via WeChat message.

There will be no specific training session provided to the users. After successfully registering as participants in the study, participants will receive a message to introduce the versions of the program they are going to use in the trial. As the program was built natively on the WeChat platform by adapting the WeChat mini-program design guideline, WeChat users are expected to have no challenges in using any functions of the program.

### Compensation

Participants in both groups will receive weekly WeChat messages to remind them to enter their daily cigarette consumption. The compensation for participation was delivered in a form called WeChat red packet (*Hongbao*). In Chinese culture, red packet is a monetary gift that is given during holidays or special occasions such as weddings, graduation, or the birth of a baby [28]. The red color of the envelope symbolizes good luck and is a symbol to ward off evil spirits [28]. The WeChat red packet is a widely used online currency in China and is transferrable through the WeChat platform [28]. Recipients of the WeChat red packet can use the value of the red packet to purchase goods or use services in China [29]. The WeChat red packet will be provided to participants as compensation to enter the data as requested. Participants from both intervention and control groups will receive the same reminding message and the same WeChat red packet. There is no specific incentive for a particular group to provide their smoking status.

The compensation in this study will be given through 7 sessions (registration and 6 weekly data collection of cigarette consumption in the past week). Once participants complete each data entry session, the WeChat red packet pops out as compensation (participants in both groups receive the same WeChat red packet). The answers to the questionnaires had no impact on the value or form of the WeChat red packet participants (in both groups) received.

It has been proven that incentives may be able to give a useful improvement in reducing the quantity of missing data in trials [30-33]. A small financial incentive has minimal impact on the trial’s results [34]. In this trial, the financial value of the WeChat red packet provided to each participant (both groups) was ¥35 RMB (approximately US $4.93), which is considered as a small financial incentive [34]. Meanwhile, compared with traditional methods of incentive delivery (eg, courier post and cash), the WeChat red packet is more secure and convenient. The WeChat red packet was only used as a method to maintain the completion of questionnaires and retention of the trial. Upon completion of the 6-week follow-up, the full version of the SCAMPI program will be offered to participants in the control group.

### Procedures

**Figure 1:**
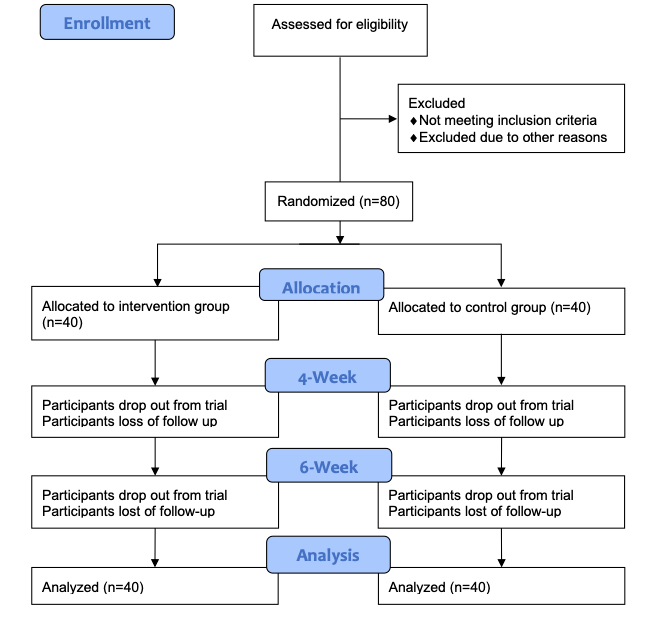
User experience in the trial.

The flow of participants’ experience in the trial is presented in Figure 1. After providing informed consent, participants will be randomly allocated to different groups (intervention and control group, n=40 in each group). Participants randomized to the intervention group will have access for 6 weeks to the full version of the SCAMPI program, and participants randomized to the control group will have access for 6 weeks to the restricted version of the SCAMPI program. On completion of the 6-week follow-up assessment, participants in the control group will be offered free full access to the SCAMPI program. All data on program use were recorded until the end of the trial.

All participation and procedures of the trial will be completed online through the WeChat platform. Participants who report themselves has 30-day continuous smoking abstinence at 6-week follow-up will receive a pack of cotinine test kit to test the nicotine levels of their saliva to biochemically verify their self-reported smoking abstinence.

Data of the trial will be collected online via the WeChat platform. Participants in both groups recorded their daily cigarette consumption for the week throughout the trial period. Usage data to the corresponding versions of the SCAMPI program were recorded by the server of the program. Participants’ baseline data and satisfaction with the program (for participants in the intervention group only) were collected through electronic questionnaires (registration questionnaire and end-of-trial questionnaire).

The registration questionnaire asked participants about their demographics, smoking status, willingness to quit smoking, and so on (Multimedia Appendix 4). The registration questionnaire was developed based on a national online survey about smoking behaviors of Chinese smokers [5,27]. The end-of-trial questionnaire (Multimedia Appendix 5) was developed and shaped based on a standard questionnaire, the MARS questionnaire [26]. It focuses on assessing users’ perception and satisfaction with the app or software or mobile program they used. Both questionnaires were overviewed and commended by experts from WeChat to ensure that their user experience met the Chinese users’ preferences.

### Retention

To prevent missing data, an effort will be made to retain the participants in the trial for the follow-up data collection. However, participants who withdraw their consent will discontinue their participation in the study. Participants who continue not entering their smoking status for 2 weeks will be considered as lost to follow-up. Participants who dropped out of the trial or who were lost to follow-up were considered to be smoking.

The strategies for improving the adherence to the study include the following: (1) gentle WeChat messages reminder—a weekly message will be sent to participants in both groups to remind them to enter the data about their daily cigarette consumption of the week, (2) participants in both groups will receive a WeChat red packet after they enter the data as requested, and (3) a button for *feedback* is available to participants in both groups on the main page of the SCAMPI OA. Participants were prompted to use text or voice messages to make their feedback or report their usage difficulties. Instant guidance to support participants to use the program will be provided by the program.

### Withdrawal Criteria

As part of the informed consent procedure and in accordance with best practice guidelines, the participant information and consent processes will clearly state that participation is voluntary, and participants will be free to withdraw at any stage of the research. Other reasons for withdrawal include the termination of the study for any reason.

As a mobile social network–based smoking cessation intervention, we believe that the intervention is barely harmful to any users. If a participant reports a severe physical or mental discomfort by using the SCAMPI program, the investigator (JC) will terminate their participation immediately and refer them to health care professionals. Events will be recorded and reported.

## Results

### Baseline Assessment

At the baseline assessment, the following data will be collected: age, marital status, employment status, family structure, age at smoking initiation, smoking cessation history, smoking status (consumption and frequency), and smoking cessation services usage history. These data will be collected at the beginning of the trial using an electronic questionnaire called the registration questionnaire (Multimedia Appendix 5).

### Primary Outcome Measure

The primary outcome of this pilot trial will be the participants’ biochemical verified 30-day smoking abstinence at 6-week follow-up. At the 6-week time point, researchers will review participants’ (in both groups) self-reported smoking status in the past 30 days to identify their self-reported 30-day smoking abstinence. The 30-day smoking abstinence is usually used as the primary outcome measure for smoking cessation intervention trials recommended by the Society for Research on Nicotine and Tobacco [35]. Quit failure was defined as any cigarettes smoked in the past 30 consecutive days. This measurement is also known as the 10th level of smoking abstinence in the Chinese standard for smoking cessation [23].

At the 6-week time point, a nicotine cotinine saliva test kit will be sent to participants (in both groups) who report themselves with 30-day smoking abstinence to verify their smoking abstinence status. These participants will be requested to complete the test at home (clear instruction provided) and upload a photo or a short video of the strip test results to the SCAMPI OA. The cotinine test has proven to be a valid and reliable method to test saliva samples for verification of smoking status [36,37].

### Secondary Outcome Measures

The secondary outcomes of the study included participants’ tobacco consumption changes through the trial period, retention of the trial, program (both versions) usage, and users’ satisfaction with the program (only for intervention group participants).

Tobacco consumption changes include measures of the following:

1. Participants’ cigarette consumption reduction: Participants will be required to provide their daily cigarette consumption (as a categorial range such as “1 to 5 sticks cigarettes per day”) at the baseline assessment. By using these data to compare with their daily smoking status through the pilot trial, researchers will be able to identify the reduction in cigarette consumption of participants (in both groups). Participants who reduce their consumption by at least one category (eg, from “16 to 20 sticks of cigarettes per day” to “10 to 15 cigarettes per day”) from baseline will be counted as successfully achieving smoking reduction.
2. 7-day smoking abstinence at 4-week and 6-week follow-up: At the 4-week and 6-week time points, researchers reviewed and analyzed participants’ (in both groups) smoking status in the past 7 days to identify their 7-day smoking abstinence at both these time points. A 7-day smoking abstinence is defined as not a single cigarette being smoked in the past 7 days [35].
3. 30-day self-reported smoking abstinence at 6-week follow-up: At the 6-week time point, researchers will review and analyze participants’ smoking status in the past 30 days to identify their self-reported 30-day smoking abstinence. A 30-day smoking abstinence is defined as not a single cigarette being smoked in the past 30 days [35].

The retention rate of the trial refers to participants providing their daily smoking status at the end of every week.

Program usage will be measured by the number of times participants interact with the assigned versions of the SCAMPI program. At the end of the pilot trial, researchers will review the data on how many times participants like, comment, forward, post content from SCAMPI OA on a personal board, or have a conversation with their friend. The data on the number of times participants used the SCAMPI mini-program (for participants in the intervention group only) and the data on the number of times participants (in both groups) communicated with the program (by sending text messages, voice messages, or emoji) to the SCAMPI OA will also be collected and reviewed. These data may reflect the level of engagement between users in the SCAMPI program.

Users’ satisfaction with the SCAMPI program will be measured by a standard MARS questionnaire at the end of the trial. As some questions on the questionnaire are not applicable to the restricted version of the program, only participants in the intervention group will be requested to complete the questionnaire (Multimedia Appendix 6) [38,39].

### Data Collection

All data of the study will be collected online through the social network platform WeChat. Table 3 shows the schedule of different data collections of the study.

**Table 3:** Schedule of study data collection.

Characteristics		Week 1 (day 0)	Week 1-5 (day 1-35)	Week 6 (day 36-42)
		Screening + baseline data collection + randomization	Follow-up data collection	Follow-up data collection
**General data**				
	Eligibility	✓^a^	—^b^	—
	Electronic consent	✓	—	—
	Demographics	✓	—	—
	Quitting history	✓	—	—
	Smoking cessation interventions usage	✓	—	—
**Primary outcome**				
	Participants’ biochemical verified 30-day smoking abstinence at 6-week follow-up	—	✓	✓
**Secondary outcomes**				
	Cigarette consumption	✓	✓	✓
	7-day smoking abstinence at 4-week and 6-week follow-up	—	✓	✓
	30-day self-reported smoking abstinence at 6-week follow-up	—	✓	✓
	Participants’ retention	✓	✓	✓
	Program usage	—	✓	✓
	Users’ satisfaction with the program (intervention group)	—	—	✓

^a^Data were collected in the described timepoint.

^b^Data were not collected at the described timepoint.

Screening data will be collected at week 1 (day 0) via the registration questionnaire to determine eligibility for all individuals who read the study advertisement and entered the study registration system. The questionnaire consists of a short overview of the aim of the study and questions regarding the person’s demographics, additions, quitting history, willingness to quit, and the usage of other smoking cessation interventions. All questionnaires of the trial were delivered by the Tencent Questionnaire platform, which is compatible to the WeChat platform.

Data on participants’ cigarette consumption will be collected from day 1 to day 42 of the study via the same questionnaire platform. At the end of each week, participants will be provided with a link through WeChat messages to have access to the corresponding online questionnaire to enter their cigarette consumption status on each day of the week.

Users’ satisfaction with the SCAMPI program (intervention group only) will be collected on day 42 along with the last weekly cigarette consumption status checking questionnaire. Data collected through online questionnaires will be downloaded from the platform and stored in a secure university server after every collection.

The program usage data (both versions) will be recorded by the WeChat mini-program and OA databases. These data will be exported and stored in the same place as data collected from online questionnaires.

On day 42, a link of ShunFeng courier WeChat mini-program will be sent to participants who self-reported themselves as 30-day smoking abstinence. Participants who received the link were requested to provide their address and recipient details to the courier through WeChat (none of these data were accessible or recorded by the research team). On the basis of the provided details, the kits with instructions will be sent to participants. Participants were requested to use the kit as instructed, take a photo or shoot a short video about how they used the kits and the relevant results (personal images were not asked), and send the test results to the SCAMPI OA.

### Data Management

The National Institute for Health Innovation, University of Auckland, is responsible for storing and protecting the research data. As a digital trial, all data will be entered electronically. All electronic data will be stored on the university computer with password protection. Only the principal investigator and members of the research team will have access to the computer-based data. All participants will be assigned a specific code number to protect their confidentiality. All data will be under the unique code number and stored for a period of 3 years after completion of the study.

### Statistical Methods

Data from this study will be exported from different databases, the WeChat mini-program and OA databases for the data related to the SCAMPI program usage and the Tencent Questionnaire database for the data related to participants’ demographics, additions, quitting history, willingness to quit, the usage of other smoking cessation interventions, and cigarette consumption during the study.

Baseline data collected from all participants will be summarized and presented. Continuous variables (eg, age) will be presented as numbers observed, means, and standard deviations. Categorical variables (eg, marital status) will be presented as frequencies and percentages. As any differences between randomized groups at baseline could only have occurred by chance, no formal significance testing will be conducted. Primary and secondary outcomes will be summarized descriptively at the identified time points. A generalized linear mixed model will be used to assess the effect of the SCAMPI program on participants’ smoking cessation outcomes.

### Power

We determined the power for the proposed main trial of this study. A total sample size of 530 (265 per group) will have 90% power at 5% significance (two-sided) to detect an absolute difference of 10% on the primary outcome between the 2 groups, assuming a control rate of 6.7% [19] and 20% loss to follow-up. Given the budget and resource limitations, in this study, we aim to recruit around (80/530, 15.1%) of the total sample size required for the main trial, that is, n=80 (40 per group), using an open recruitment strategy to recruit all participants within a month. At the end of the study, we will re-estimate the power of the final percentage differences between groups in the effectiveness report to further determine the exact final sample size of a full trial. The sample size is considered as a major limitation of the study.

### Ethics Approvals

Ethics approval (reference number: 021649) was obtained from the University of Auckland Human Participants Ethics Committee. As this study targets Chinese male smokers, ethics approval (reference number: ZGL201801-2) was obtained from the Zhejiang University School of Public Health Research Ethics Committee.

### Consent

All eligible individuals are given an electronic copy of the information sheet and informed consent form to read via WeChat. The information sheet provides a summary of the study, and the informed consent document states what the individual is about to participate in, the individual’s rights as a research participant, and information about confidentiality. Individuals are encouraged to communicate with researchers through SCAMPI OA if they need any further explanation or information about the study. If the person chooses to participate in the study, that person will be asked to tap the *agree* button on the informed consent page of the registration questionnaire (Multimedia Appendix 5). Subjects who refuse to participate or who withdraw from the study were treated without prejudice. The reason for refusal or withdrawal will be noted if reported.

### Confidentiality

All study-related data will be exported and stored securely at the university server under a coded identification number to maintain participant confidentiality. All data that contain personal identifiers, such as screening data for eligibility and informed consent forms, will be stored separately from study records identified by a code number. All local databases were secured with a password-protected access system. All data of the study were collected and stored electronically.

## Discussion

### Principal Findings

This pilot trial and its findings will contribute to the evidence available to inform the development and implementation of the SCAMPI program as a mobile social network–based smoking cessation tool. The primary outcome of the study was to contribute to an understanding of the preliminary effectiveness of the SCAMPI program as a smoking cessation intervention for the target population. The overall implementation of the study can have an impact on delivering smoking cessation interventions through social network platforms such as WeChat. The use of the SCAMPI program is not restricted to any specific place, time, or situation, and it is free to use. It can also contribute to the reduction of health care costs. In addition, the study will contribute significantly to future research on social network–based health intervention development and evaluation. It may provide new ideas about applying social network platforms to improve recruitment efficiency and engagement in public health research projects. The relatively small sample size and short-term follow-up due to time and budget limitations are common with mobile smoking cessation trials [40], which is a threat to the quality of the trial.

### Trial Status

Recruitment for this project commenced in January 2019 and was completed in the same month. Follow-up data collection commenced and was completed by the end of June 2019.

## Appendix

Multimedia Appendix 1 Development Questionnaire.

Multimedia Appendix 2 Usability Testing Questionnaire.

Multimedia Appendix 3 Transition from behavioral factors to behavior change techniques.

Multimedia Appendix 4 Screenshots and Quick Response codes of the program.

Multimedia Appendix 5 Registration Questionnaire.

Multimedia Appendix 6 End-of-trial Questionnaire.

## Abbreviations

BCT: behavior change techniques
BCW: behavior change wheel
CCSCG: China Clinical Smoking Cessation Guideline
COM-B: capability, opportunity, and motivation to behavior
CPD: collaborative product development
MARS: Mobile App Rating Scale
OA: official account
QR: Quick Response
RCT: randomized controlled trial
UCD: user-centered design
UI: user interface
UX: user experience
