# Supplementary material for: A Mobile Social Network–Based Smoking Cessation Intervention for Chinese Male Smokers: Protocol for a Pilot Randomized Controlled Trial
Source: JMIR Res Protoc. 2020 Sep 18;9(9):e18071. doi: 10.2196/18071 (PMC7532454; doi:10.2196/18071)
Supplement: Multimedia Appendix 1 [file resprot_v9i9e18071_app1.docx]

Multimedia Appendix 1: Development Questionnaire

1. Your year of birth is:

1974 ~ 1993

2. Please select the city you currently live in:

3. Your current occupation is:

|  | Student |
| --- | --- |
|  | Businessman |
|  | Researcher |
|  | Engineer |
|  | Technician |
|  | Government Worker |
|  | Other (Please specify) |

4. Your current marital status is:

|  | Single |
| --- | --- |
|  | In a relationship |
|  | Married |
|  | Divorced |

5. You have ____ child / children

|  | None |
| --- | --- |
|  | One |
|  | Two |
|  | More than 2 |

6. You started smoking when you were

|  | Under 18 |
| --- | --- |
|  | 18~25 years |
|  | 26~30 years |
|  | 31~40 years |
|  | 41~44 years |

7. Have you tried to quit smoking?

|  | Yes (to Question 8) |
| --- | --- |
|  | No (to Question 10) |

8. How many times have you tried to quit smoking?

|  | Once |
| --- | --- |
|  | Twice |
|  | Three times |
|  | Four times |
|  | Five times |
|  | More than 5 times |

9. The longest time you had stopped smoking lasted for ____month(s)

|  | Less than 1 month (please specify how many days) |
| --- | --- |
|  | 1-3 months |
|  | 4-6 months |
|  | 7-9 months |
|  | 10-12 months |
|  | More than 12 months |

10. Are you a daily smoker?

|  | Yes (to Question 12) |
| --- | --- |
|  | No (to Question 11) |

11. How often do you smoke?

|  | Once every two days |
| --- | --- |
|  | Once every three days |
|  | Once every four days |
|  | Once every five days |
|  | Once every six days |
|  | Once every seven days |

12. On average, how many cigarettes you smoke per day?

|  | 1-5 stick(s) |
| --- | --- |
|  | 6-10 sticks |
|  | 11-15 sticks |
|  | 16-20 sticks |
|  | 21-25 sticks |
|  | 26-30 sticks |
|  | 31-35 sticks |
|  | 36-40 sticks |
|  | More than 40 sticks |

13. Please select your preferred cigarettes.

|  | China made |
| --- | --- |
|  | Imported |

14. Please select the type of cigarettes you usually smoke.

|  | Manufactured cigarettes |
| --- | --- |
|  | Roll-your-own cigarettes |
|  | Tobacco leaf with traditional devices |
|  | Others |

15. Have you ever used / currently using any stop smoking apps?

|  | Yes (to Question 16) |
| --- | --- |
|  | No (to Question 18) |

16. Please list the stop smoking apps you have ever used?

17. How useful do you think were the stop smoking apps you have used / currently using?

|  | Not useful at all |
| --- | --- |
|  | Not very useful |
|  | Neither useful nor not useful |
|  | Fairly useful |
|  | Very useful |

18. Why do you not use mobile stop smoking apps (MCQ)

|  | Never thought about it |
| --- | --- |
|  | Did not know mobile smoking cessation apps were available |
|  | Do not feel mobile smoking cessation apps are useful |
|  | Other reasons (never smoking in routine) |

19. What would be the main reasons for you to use a smoking cessation app? (MCQ)

|  | To know more about the harms from smoking |
| --- | --- |
|  | To cut down the number of cigarettes I smoke |
|  | To quit smoking completely |
|  | To save money I currently spend on cigarettes |
|  | For my family’s health |
|  | Other reasons |

20. Please identify the main factors that usually trigger you to smoke (MCQ)

|  | In social situations (in workplace or business situations with friends, colleagues, etc.) |
| --- | --- |
|  | After a meal |
|  | Feeling depressed or down in mood |
|  | Feeling positive or happy |
|  | Feeling stressed or anxious |
|  | Feeling tired |
|  | During entertainment (e.g. playing cards, Majiang, watching sports, etc.) |
|  | Reading or Writing |
|  | When alone |
|  | When drinking alcohol |
|  | Other situations |

21. Please identify the main factors that could motivate you to quit smoking / use a stop smoking app. (MCQ)

|  | Personal health concerns |
| --- | --- |
|  | Family health concerns |
|  | High cost of cigarettes |
|  | Family pressure (wife / children stop you smoke) |
|  | Advice and examples from family / friends |
|  | Advice from doctors or other health professionals |
|  | Restrictions on smoking (in work place, on public transportation, at home) |
|  | Social stigma of smoking |
|  | Don’t want child / children growing up with smoking |
|  | Others |

22. Please identify may prevent you trying to successfully quit smoking. (MCQ)

|  | Exposure to triggering conditions (e.g. given a gift of cigarettes by someone) |
| --- | --- |
|  | Lack of knowledge of the risk of harms to my health from smoking |
|  | Lack of knowledge about how to cope with craving and other withdrawal symptoms |
|  | Lack of knowledge of the government laws and policies related to tobacco control |
|  | I enjoy smoking |
|  | It is too much of a habit, so is hard to stop smoking |
|  | I don’t have any other methods to deal with unwanted emotions |
|  | Others |

23. Please identify the functions that you think a smoking cessation app should have (MCQ)

|  | Calculator: Tracks dollars saved and health benefits accrued over time since quitting |
| --- | --- |
|  | Calendar: Tracks days until and after the quit date |
|  | Game: Provide games to distract your craving |
|  | Practical tools: Use hypnosis/meditation techniques to stop smoking |
|  | Informational: Provide information on quitting smoking |
|  | Health tester: Help you to test your health conditions (lung, heart, oesophagus and other organs’ health levels) |
|  | Rationing: limits the numbers of cigarettes and / or the time in which cigarettes could be smoked |
|  | Planning: Helps you to design and implement a quitting plan |
|  | Social network: Provides a social network with other smokers who want to quit for sharing |
|  | Notification: quitting experience, health professionals will also provide quitting advice in this network |
|  | Notifying: Notifies your quitting plan and progress |
|  | GPS function: Reminds you whether you are at tobacco control areas |
|  | Others |
