# Supplementary material for: A Mobile Social Network–Based Smoking Cessation Intervention for Chinese Male Smokers: Protocol for a Pilot Randomized Controlled Trial
Source: JMIR Res Protoc. 2020 Sep 18;9(9):e18071. doi: 10.2196/18071 (PMC7532454; doi:10.2196/18071)
Supplement: Multimedia Appendix 3 [file resprot_v9i9e18071_app3.docx]

Multimedia Appendix 3: Transition from Behavioural Factors to BCTs

| **Behavioural factors** | **COM-B components** | **BCTs code** | **Description and examples on SCAMPI** | **1** | **2** |
| --- | --- | --- | --- | --- | --- |
| 1. Lack of methods to cope with unwanted emotion  2. Poor health literacy of smoking harms and SHS harms  3. Poor health literacy of cessation benefits to self and family  4. Lack of methods to quit smoking  5. Poor awareness about existing smoking cessation services | Psychological Capability | BM1 | Provide information on consequences of smoking and smoking cessation  E.g. messages of smoking harms | √ | √ |
|  |  | BM2 | Boost motivation and self-efficacy  E.g. motivating messages when users about to have a slip of relapse | √ | √ |
|  |  | BM3 | Provide feedback on current behaviour  E.g. remind users how many days they had achieved smoke free | √ | √ |
|  |  | BM4 | Provide rewards contingent on successfully stopping smoking  E.g. praise users when he achieved one-week smoke free | √ | √ |
|  |  | BM5 | Provide normative information about others' behaviour and experiences  E.g. smoke free board shows how others are doing | √ | √ |
|  |  | BM10 | Explain the importance of abrupt cessation  E.g. provide information about even one cigarette harms | √ | √ |
|  |  | BS1 | Facilitate barrier identification and problem solving  E.g. provide advice to help users overcome unwanted emotion which is considered as one key factor to trigger smoking | √ | √ |
|  |  | BS2 | Facilitate relapse prevention and coping  E.g. provide information about how lapses occur and how they lead to relapse and to develop specific strategies for preventing lapses | √ | √ |
|  |  | BS3 | Facilitate action planning/develop treatment plan  E.g. the programme works with users to generate clear quit plans | √ | √ |
|  |  | BS4 | Facilitate goal setting  E.g. “30-day smoke free challenge” - quit date and goals that support the aim of remaining abstinent | √ | √ |
|  |  | BS5 | Prompt review of goals  E.g. prompt users to review how far the he has achieved the goal of abstinence | √ | √ |
|  |  | BS6 | Prompt self-recording  E.g. the programme provides function for users to record and review potentially useful information | √ | √ |
|  |  | BS9 | Set graded tasks  E.g. from providing daily smoke free data to taking 30-day smoke free challenge | √ | √ |
|  |  | BS10 | Advise on conserving mental resources  E.g. advise on ways of minimising stress and access to relevant resources (focus on avoiding unwanted emotions) | √ | √ |
|  |  | A5 | Give options for additional and later support  E.g. provide links to other smoking cessation services those were available | √ | √ |
|  |  | RC2 | Elicit and answer questions  E.g. the programme allows users to ask questions whenever they want and aims to answer as soon as possible | √ | √ |
|  |  | RC5 | Offer/direct towards appropriate written materials  E.g. materials and information shown on the programme will be clearly identified their sources | √ | √ |
|  |  | RC6 | Provide information on withdrawal symptoms  E.g. information about what are, and are not, nicotine withdrawal symptoms, and so on will be provided | √ | √ |
|  |  | RC9 | Summarise information/confirm client decisions  E.g. terms and conditions, as well as participation information sheet will be provided before users participate and use the SCAMPI programme | √ | √ |
|  |  | RC10 | Provide reassurance  E.g. provide information about users’ experience are normal and expected, positive feedback to encourage continual abstinence after using the programme | √ | √ |
| 6. Problematic impression of smoking behaviour (e.g. cool) | Reflective Motivation | BM6 | Prompt commitment from the client there and then  E.g. users need to assure that if they want to take the challenge of being smoke free for period of time | √ | √ |
|  |  | BM7 | Provide rewards contingent on effort or progress  E.g. programme gives praise for the effort and progress the user is making toward their goal of abstinence | √ | √ |
|  |  | BM8 | Strengthen ex-smoker identity  E.g. provide information about the importance of regarding smoking as something that is ‘not an option’, including the ‘not a puff’ | √ | √ |
|  |  | BM9 | Identify reasons for wanting and not wanting to stop smoking  E.g. this had been accomplished in development questionnaire 1 by identifying reasons of quitting and factors that may prevent successful cessation | √ | √ |
|  |  | BM11 | Measure CO  E.g. this is not deliverable by the nature of mHealth app-based smoking cessation interventions | √ | X |
|  |  | RC8 | Elicit client views  E.g. this had also been achieved by development questionnaire 1 by asking users’ view on smoking and smoking cessation, as well as their experience on other cessation services | √ | √ |
|  | Automatic Motivation | BS10 | Advise on conserving mental resources  E.g. provide advice on methods to minimise stress and access to other resources for mental health services (focus on avoiding using smoking as a method to cope from unwanted emotions) | √ | √ |
| 7. High chance to be in triggering environment (e.g. gifted with cigarettes, peer smoking, etc.)  8. Low cost of tobacco | Physical Opportunity | BS7 | Advise on changing routine  E.g. provide advice on changing routine to minimise exposure to smoking cues | √ | √ |
|  |  | BS8 | Advise on environmental restructuring  E.g. provide advice on ways of changing the physical environment to minimise exposure to smoking cues | √ | √ |
|  |  | A1 | Advise on stop-smoking medication | NA | NA |
|  |  | A3 | Adopt appropriate local procedures to enable clients to obtain free medication | NA | NA |
|  |  | A4 | Ask about experience of stop smoking medication that the smokers is using | NA | NA |
| 9. Using smoking as a social communication tool (e.g. peer smoking and sharing cigarettes) | Social Opportunity | BS11 | Advise on avoiding social cues for smoking  E.g. provide advice on encouraging smoking peer to quit smoking together to minimise chance of exposing to peer smoking situation | √ | √ |
|  |  | A2 | Advise on/facilitate use of social support  E.g. virtual peer support group will be built on the programme to encourage communication and peer support between programme users | √ | √ |
| 10. Perceive smoking as an emotion coping tool: unwanted emotion triggers smoking behaviour  11. Perceive smoking as an entertainment tool: entertainment (e.g. playing cards) triggers smoking behaviour | Programme Design & Development | RD1 | Tailor interactions appropriately  E.g. information provided by users (e.g. cost of cigarettes smoke) will be used to tailor intervention (cost saved from not smoking) | √ | √ |
|  |  | RD2 | Emphasise choice  E.g. provide evidence and practice-based choices for users to adapt to keep themselves away from smoking | √ | √ |
|  |  | RI1 | Assess current and past smoking behaviour  E.g. users’ smoking behaviour will be monitored before, during and after using the programme | √ | √ |
|  |  | RI2 | Assess current readiness and ability to quit  E.g. users’ readiness and ability to quit will be assessed before using the programme | √ | √ |
|  |  | RI3 | Assess past history of quit attempts  E.g. users’ history of quit attempts will be assessed before using the programme | √ | √ |
|  |  | RI4 | Assess withdrawal symptoms  E.g. users will be prompted to report their withdrawal symptoms once they have any during using the programme | √ | √ |
|  |  | RC1 | Build general rapport  E.g. users mother language (Chinese) will be used and used in a comfortable and manner concerned way to ensure the development of friendly and professional relationship built between the programme and users | √ | √ |
|  |  | RC3 | Explain the purpose of CO monitoring  Since it is undeliverable by mHealth app-based intervention, the BCT is not applicable to SCAMPI | NA | NA |
|  |  | RC4 | Explain expectations regarding treatment programme  E.g. information about the programme, what it involves, and what it requires of the users will be clearly indicated before users using it | √ | √ |
|  |  | RC7 | Use reflective listening  E.g. users will be allowed to communicate with the programme whenever they want, some pre-set responses will be provided for general questions, while manual supports will be provided for specific issues | √ | √ |

1: Assessment by the China clinical smoking cessation guideline

2: Assessment by the WeChat mini-programme design guideline

NA: Not applicable
