# Supplementary material for: A Mobile Social Network–Based Smoking Cessation Intervention for Chinese Male Smokers: Protocol for a Pilot Randomized Controlled Trial
Source: JMIR Res Protoc. 2020 Sep 18;9(9):e18071. doi: 10.2196/18071 (PMC7532454; doi:10.2196/18071)
Supplement: Multimedia Appendix 5 [file resprot_v9i9e18071_app5.docx]

Multimedia Appendix 5: Registration Questionnaire

Hello, this is a research project run by Tencent and the University of Auckland. The main intention of this study is to identify the preliminary effectiveness of the SCAMPI programme (a smoking cessation intervention) through a pilot randomised controlled trial. This study will focus on male smokers only.

If you meet the participants requirements, welcome to be one of the study participants. Your compensation for participation will be released as WeChat Red Packet in a weekly basis.

Note:
1. Please send “complete” to the SCAMPI official account “NIHISCAMPI” after you are finishing the register questionnaire, or your questionnaire will be deleted.
2. Please don’t change your WeChat account name during the trial period, or please inform the SCAMPI official account “NIHISCAMPI” if you want to change your WeChat account name.

**Participants requirements:**

-  Chinese male smokers aged 25 to 44 years

-  Have strong intention to quit smoking

**Your involvement:**

-  Fill questionnaire

-  Using the SCAMPI programme

-  Receiving quitting information

-  Providing smoking status to receive red packet compensation (once a week, total value ¥35 RMB)

-  Out of lab saliva testing (you will be requested to provide a photograph or shot video)

-  Fill an end-of-trial questionnaire

**Safety and information:**

We do not anticipate any risks to participants in this project. However, if you experience discomfort, please contact:
Zhejiang University Centre for Tobacco Control Research
Address: No.25 Zijing Gang Campus, Zhejiang University

Contact: 0571-88208219
Website: www.tfcampuschina.com
All information collected from this project will not contain any identifying information. If any, they will be anonymised. When the results of this project are written up or reported in any context, no material that could personally identify you will be used.

**WeChat red packet (compensation):**

You will receive ¥35 RMB as participation compensation of the trial. Your compensation will be delivered in the form of WeChat red packet each time after you provide information to the requested e-questionnaire. Your answers to the e-questionnaire will have no impact on the red packet you receive.

**Project results:**

The project will be run over a period until April 2019. We anticipate that the results will be available and published on relevant academic journals. We will also provide a link to the publication for reviewing and reading on the SCAMPI official account.

**Contact:**

Jinsong Chen
PhD candidate,
National Institute for Health Innovation, The University of Auckland
Email: jinsong.chen@auckland.ac.nz
*This project had been approved by:*The University of Auckland Human Participants Ethics Committee (Reference No.: 021649) Email: [ro-ethics@auckland.ac.nz](mailto:ro-ethics@auckland.ac.nz)

The Zhejiang University School of Population Health Medical Ethics Committee (Reference No.: ZGL201801 - 2)

**Terms and conditions:**

(you can take screenshot to save the information in this form)

- I agree to participate this project
- I have been informed that I can withdraw from the project by anytime I want without any reasons (by then except already saved information, all my participation information will be deleted (although you will lose your opportunity to receive your participating compensation)
- I realise I will be asked about my smoking status (in weekly basis) and information in related to my smoking behaviour
- I know all information about my personal identity will not be collected or published

Note:
This project is run by the National Institute of Health Innovation, which is responding to all legal responsibility. Tencent is not the organiser nor operator. Tencent will not take any legal responsibility in regarding to this project.

I had read and understand all information about, I agree to take part in this research project.

Agree

Disagree

1. Please identify your gender

|  | Male |
| --- | --- |
|  | Female (sorry you did not meet the participant inclusion criteria, you will not be able to take part in this project, thanks for your support) |

2. Which age groups are you belonging in?

|  | 0-24 (sorry you did not meet the participant inclusion criteria, you will not be able to take part in this project, thanks for your support) |
| --- | --- |
|  | 25-44 |
|  | 45-64 (sorry you did not meet the participant inclusion criteria, you will not be able to take part in this project, thanks for your support) |
|  | 65+ (sorry you did not meet the participant inclusion criteria, you will not be able to take part in this project, thanks for your support) |

3. Are you a current smoker?

|  | Yes |
| --- | --- |
|  | No (sorry you did not meet the participant inclusion criteria, you will not be able to take part in this project, thanks for your support) |

4. Do you currently receiving any type of smoking cessation services

|  | Yes (sorry you did not meet the participant inclusion criteria, you will not be able to take part in this project, thanks for your support) |
| --- | --- |
|  | No |

5. Please identify your willingness to quit smoking

|  | 1 (totally unwilling to quit) |
| --- | --- |
|  | 2 |
|  | 3 |
|  | 4 |
|  | 5 (strongly willing to quit) |

**General Information**

6. You started smoking when you were

|  | Under 18 |
| --- | --- |
|  | 18~25 years |
|  | 26~30 years |
|  | 31~40 years |
|  | 41~44 years |

7. Are you a daily smoker?

|  | Yes (to Question 8) |
| --- | --- |
|  | No (to Question 9) |

8. On average, how many cigarettes you smoke per day? (to question 10)

|  | 1-5 stick(s) |
| --- | --- |
|  | 6-10 sticks |
|  | 11-15 sticks |
|  | 16-20 sticks |
|  | 21-25 sticks |
|  | 26-30 sticks |
|  | 31-35 sticks |
|  | 36-40 sticks |
|  | More than 40 sticks |

9. How often do you smoke?

|  | Once every two days |
| --- | --- |
|  | Once every three days |
|  | Once every four days |
|  | Once every five days |
|  | Once every six days |
|  | Once every seven days |

10. Have you tried to quit smoking?

|  | Yes (to Question 12) |
| --- | --- |
|  | No (to Question 17) |

11. How many times have you tried to quit smoking?

|  | Once |
| --- | --- |
|  | Twice |
|  | Three times |
|  | Four times |
|  | Five times |
|  | More than 5 times |

12. The longest time you had stopped smoking lasted for ____month(s)

|  | Less than 1 month (please specify how many days) |
| --- | --- |
|  | 1-3 months |
|  | 4-6 months |
|  | 7-9 months |
|  | 10-12 months |
|  | More than 12 months |

13. Have you ever used any stop smoking apps?

|  | Yes (to Question 16) |
| --- | --- |
|  | No (to Question 18) |

14. Which types of the following smoking cessation service had you ever received? (MCQ)

|  | Smoking cessation clinic |
| --- | --- |
|  | QuitLine |
|  | Smoking cessation medication |
|  | Smoking cessation text messaging services |
|  | Smoking cessation apps |
|  | Other (please specify) |

15. Do you think the smoking cessation services you received are helpful?

|  | 1 not helpful at all |
| --- | --- |
|  | 2 |
|  | 3 |
|  | 4 |
|  | 5 very helpful |

16. Why don’t you use smoking cessation services (MCQ)

|  | Did not know mobile smoking cessation apps were available |
| --- | --- |
|  | Do not feel mobile smoking cessation apps are useful |
|  | Never thought about it |
|  | Other reasons (please specify) |

17. Please identify the main factors that usually trigger you to smoke (MCQ)

|  | In social situations (in workplace or business situations with friends, colleagues, etc.) |
| --- | --- |
|  | At (or after) a meal |
|  | Feeling depressed or down in mood |
|  | Feeling positive or happy |
|  | Feeling stressed or anxious |
|  | Feeling tired |
|  | During entertainment (e.g. playing cards, Majiang, watching sports, etc.) |
|  | Reading or Writing |
|  | At work |
|  | When alone |
|  | When drinking alcohol |
|  | Other situations |

18. Please identify the main factors that could motivate you to quit. (MCQ)

|  | Personal health concerns |
| --- | --- |
|  | Family health concerns |
|  | High cost of cigarettes |
|  | Advice and examples from family / friends |
|  | Advice from doctors or other health professionals |
|  | Restrictions on smoking (in work place, on public transportation, at home) |
|  | Social stigma of smoking |
|  | Don’t want child / children growing up with smoking |
|  | Others (please specify) |

19. Your year of birth is:

1974 ~ 1993

20. Please select the city you currently live in:

21. Your current occupation is:

|  | Student |
| --- | --- |
|  | Government worker |
|  | Businessman (office worker) |
|  | Professional (doctor/lawyer/journalist/teacher etc.) |
|  | Worker (factory worker/labour worker etc.) |
|  | Sales and services |
|  | Self-business owner |
|  | Freelancer |
|  | Farmer |
|  | Retired |
|  | Unemployed |
|  | Other (Please specify) |

22. Your current marital status is:

|  | Single |
| --- | --- |
|  | In a relationship |
|  | Married |

23. Do you have child / children

|  | Yes |
| --- | --- |
|  | No |
