# Supplementary material for: A Mobile Social Network–Based Smoking Cessation Intervention for Chinese Male Smokers: Protocol for a Pilot Randomized Controlled Trial
Source: JMIR Res Protoc. 2020 Sep 18;9(9):e18071. doi: 10.2196/18071 (PMC7532454; doi:10.2196/18071)
Supplement: Multimedia Appendix 6 [file resprot_v9i9e18071_app6.docx]

Multimedia Appendix 6: End-of-trial Questionnaire

1. Do you feel the programme is interesting?

|  | Not interesting at all |
| --- | --- |
|  | Mostly uninteresting |
|  | OK, would like to use for a brief time (< 5 minutes) |
|  | Moderately interesting; would like to use 5-10 minutes |
|  | Very interesting, would like to use repeatedly |

2. Please let us know if the programme will attract Chinese smokers (content, design and functions)?

|  | Not at all |
| --- | --- |
|  | Maybe not |
|  | Not sure |
|  | Maybe yes |
|  | Of course, yes |

3. Do you think the current smoking cessation programme has functions suitable to you?

|  | Not suitable at all |
| --- | --- |
|  | Partly not suitable |
|  | Neither suitable nor not suitable |
|  | Mostly suitable |
|  | Completely suitable |

4. Do you feel the description of the programme is easy to understand (including its icons, menu and instructions)?

|  | Not understandable or followable at all |
| --- | --- |
|  | Somewhat not understandable and followable (takes me more than half an hour to understand and follow) |
|  | Somewhat understandable and followable (takes me 15 minutes to half an hour to understand and follow |
|  | Easy to understand and follow (takes me 5 to 15 minutes to understand and follow) |
|  | The programme is very intuitive (I don't need to spend any time to understand and follow) |

5. Do you think the flow of the programme makes sense (the transitions between functions or interfaces are clear and logical)?

|  | Very unclear (I cannot see any logic at all) |
| --- | --- |
|  | Somewhat unclear (takes me more than half an hour to understand the logic) |
|  | Somewhat clear (takes me 15 minutes to half an hour to understand the logic) |
|  | Clear (takes me 5 to 15 mins to understand the logic) |
|  | Very clear (I don't need to spend any time to understand the logic) |

6. Do you feel the programme has the visually programmeropriate layout (including the size and location of icons, buttons and menu)?

|  | The layout is very bad, cannot understand at all |
| --- | --- |
|  | The layout is somewhat bad, it’s very hard to understand |
|  | The layout is neither bad nor good |
|  | The layout is somewhat good, able to understand |
|  | The layout is very good, lean and intuitive, can be understood easily |

7. How good does the programme look?

|  | Very bad, unpleasant to look at, poorly designed, clashing, mismatched colours |
| --- | --- |
|  | Somewhat bad, poorly designed, bad use of colour, visually boring |
|  | Neither pleasant, nor unpleasant |
|  | Somewhat good, pleasant, seamless graphics, consistent and professionally designed |
|  | Very good, beautiful, very attractive, memorable, stands out, use of colour enhances programme features/menus |

8. Would you recommend this programme to others?

|  | Not at all, I would not recommend this programme to anyone |
| --- | --- |
|  | Mostly not, there are very few people I would recommend this programme to |
|  | Not sure |
|  | Mostly yes, there are many people I would recommend this programme to |
|  | Definitely, I would recommend this programme to everyone |

9. Will you use this programme in the next year (about how many times)?

|  | None |
| --- | --- |
|  | 1-2 time(s) |
|  | 3-10 times |
|  | 11-50 times |
|  | more than 50 times |

10. Would you willing to pay for using this programme?

|  | Definitely not |
| --- | --- |
|  | Very unlikely |
|  | Possibly |
|  | Very likely |
|  | Definitely yes |

11. Please rate this programme.

|  | 1 (worst) |
| --- | --- |
|  | 2 |
|  | 3 |
|  | 4 |
|  | 5 (best) |

12. Please rank the programme based on your perception of its effectiveness (1 represent strongly disagree, 5 represent strongly agree)

| Questions | 1 | 2 | 3 | 4 | 5 |
| --- | --- | --- | --- | --- | --- |
| This programme has increased my awareness of the importance of quitting smoking |  |  |  |  |  |
| This programme has increased my knowledge/understanding of the need of quitting smoking |  |  |  |  |  |
| The programme has positively changed my attitudes toward quitting smoking |  |  |  |  |  |
| The programme has increased my intentions to stop smoking |  |  |  |  |  |
| This programme would encourage me to seek further help to quit smoking (if I needed it) |  |  |  |  |  |

13. Do you have any further comments about the programme, please specify
